# Inhibition of voltage-gated sodium channels by sumatriptan bioisosteres

**DOI:** 10.3389/fphar.2015.00155

**Published:** 2015-07-24

**Authors:** Roberta Carbonara, Alessia Carocci, Julien Roussel, Giuseppe Crescenzo, Canio Buonavoglia, Carlo Franchini, Giovanni Lentini, Diana Conte Camerino, Jean-François Desaphy

**Affiliations:** ^1^Section of Pharmacology, Department of Pharmacy & Drug Sciences, University of Bari Aldo MoroBari, Italy; ^2^Section of Medicinal Chemistry, Department of Pharmacy & Drug Sciences, University of Bari Aldo MoroBari, Italy; ^3^Department of Veterinary Medicine, University of Bari Aldo MoroBari, Italy

**Keywords:** pain, Nav1.7, use-dependent sodium channel block, analgesia, sumatriptan analogs, mexiletine

## Abstract

Voltage-gated sodium channels are known to play a pivotal role in perception and transmission of pain sensations. Gain-of-function mutations in the genes encoding the peripheral neuronal sodium channels, hNav1.7–1.9, cause human painful diseases. Thus while treatment of chronic pain remains an unmet clinical need, sodium channel blockers are considered as promising druggable targets. In a previous study, we evaluated the analgesic activity of sumatriptan, an agonist of serotonin 5HT_1B/D_ receptors, and some new chiral bioisosteres, using the hot plate test in the mouse. Interestingly, we observed that the analgesic effectiveness was not necessarily correlated to serotonin agonism. In this study, we evaluated whether sumatriptan and its congeners may inhibit heterologously expressed hNav1.7 sodium channels using the patch-clamp method. We show that sumatriptan blocks hNav1.7 channels only at very high, supratherapeutic concentrations. In contrast, its three analogs, namely 20b, (*R*)-31b, and (*S*)-22b, exert a dose and use-dependent sodium channel block. At 0.1 and 10 Hz stimulation frequencies, the most potent compound, (*S*)-22b, was 4.4 and 1.7 fold more potent than the well-known sodium channel blocker mexiletine. The compound induces a negative shift of voltage dependence of fast inactivation, suggesting higher affinity to the inactivated channel. Accordingly, we show that (*S*)-22b likely binds the conserved local anesthetic receptor within voltage-gated sodium channels. Combining these results with the previous ones, we hypothesize that use-dependent sodium channel blockade contributes to the analgesic activity of (*R*)-31b and (*S*)-22b. These later compounds represent promising lead compounds for the development of efficient analgesics, the mechanism of action of which may include a dual action on sodium channels and 5HT_1D_ receptors.

## Introduction

Neuropathic pain arises as a direct consequence of a lesion or disease affecting the somatosensory system (Treede et al., 2008). Voltage-gated sodium channel have a major role in the generation and conduction of the electrical pain information in the central and peripheral nervous system (Dib-Hajj et al., 2010; Catterall, 2012). The Nav1.7, Nav1.8, and Nav1.9 sodium channel isoforms, which are preferentially expressed in dorsal root ganglia (DRG) and trigeminal neurons, have been shown to be involved in physiological and pathological pain sensation (Dib-Hajj et al., 2013). Accordingly, gain-of-function mutations in the genes encoding these channels induce DRG neuron hyperexcitability and cause human painful disorders, while loss-of-function mutations of Nav1.7 cause congenital insensitivity to pain (Hoeijmakers et al., 2015). These observations strongly suggest a promising role for sodium channels as druggable targets in pain treatment (Cummins and Rush, 2007; Priest and Kaczorowski, 2007; Dib-Hajj et al., 2009; Theile and Cummins, 2011).

Treatment of neuropathic pain is an unmet medical problem, commonly characterized by resistance to conventional analgesics, such as acetaminophen, non-steroidal anti-inflammatory drugs, and low opioid doses. Currently, treatment of neuropathic pain is based on the use of tricyclic antidepressants, mixed serotonin-noradrenaline reuptake inhibitors, and antiepileptics, followed by opioids and voltage-gated sodium channel blockers (Attal et al., 2010; Jongen et al., 2013; Finnerup et al., 2015). Interestingly, a number of these drugs are able to exert, through a primary or secondary mechanism of action, a blockade of voltage-gated sodium channels (Haeseler et al., 2006; Dick et al., 2007; Wang et al., 2010b; Leﬄer et al., 2012; Stoetzer et al., 2015). We also recently demonstrated that orphenadrine, a muscle relaxant with analgesic properties, partially inhibits peripheral nerve sodium channels at clinical concentrations (Desaphy et al., 2009).

Although serotonin is considered as a critical modulator of pain transmission, its role in chronic pain is not completely understood because of the involvement of a large number of cellular targets and receptor subtypes, which may exert pro- or anti-nociceptive actions (Lopez-Garcia, 2006). Interestingly, it has been recently shown that the antinocieptive effect of serotonin on peripheral neuropathic pain is likely mediated by specific activation of 5HT_2B_ receptor (Urtikova et al., 2012). Conversely, it is widely acknowledged that the 5HT_1B/1D_ receptor agonists, such as sumatriptan, exert a selective action on cranial pain and migraine. Besides this central action, the triptans were also shown to attenuate pain-related behavior in rodent models of somatic and visceral pain, but lacked efficacy in models of peripheral neuropathic pain (Kayser et al., 2002; Nikai et al., 2008). In a previous study, we showed that sumatriptan and some newly synthesized chiral bioisosteres display analgesic activity in the mouse hot plate test (Carocci et al., 2010). Some analogs showed a greater analgesic profile compared to sumatriptan, but this activity was not necessarily correlated to serotonin agonism. Because their chemical structure contains an aryloxyethylamine backbone, which is also present in the sodium channel blocker mexiletine, we wondered whether the *in vivo* analgesic effects of sumatriptan and its novel derivatives may be related to inhibition of sodium channels expressed in the peripheral nervous system.

**FIGURE 1:**
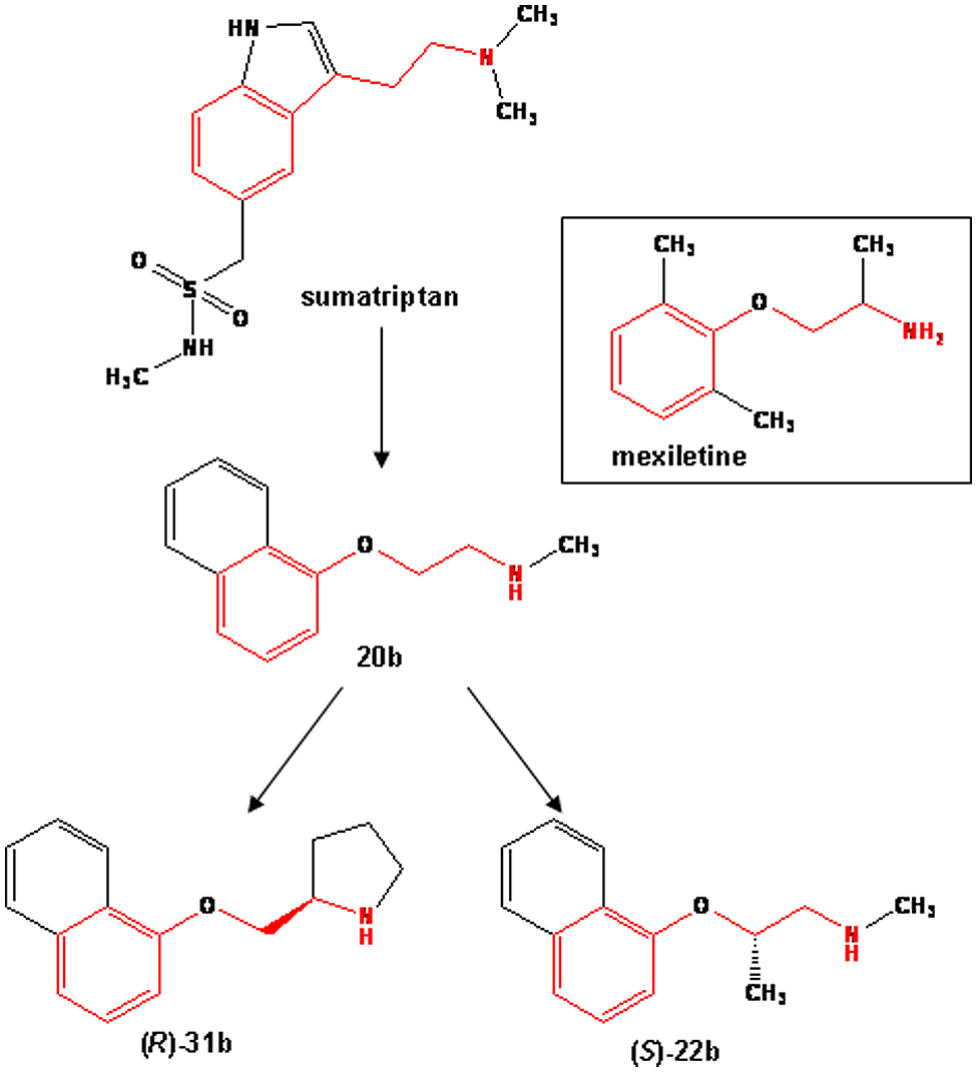
**Chemical structures of sumatriptan and its bioisosteres, compared to mexiletine.** All the compounds share a common backbone shown in red, which consists of an aromatic moiety linked to a protonable amine group through an alkyl bridge.

In this study, we evaluated the effects of sumatriptan and three of its newly synthesized analogs [namely 20b, (*R*)-31b, (*S*)-22b; **Figure 1**] on hNav1.7 functionally expressed in HEK293 cells, using the patch-clamp method. We also compared their effects on sodium currents with those of mexiletine. We found that, whereas sumatriptan inhibits sodium channels only at high, supratherapeutic concentrations, all the three analogs were more potent than mexiletine in blocking sodium channels. We further demonstrate that the most potent compound, (*S*)-22b, likely binds to the conserved local anesthetic receptor within the pore of voltage-gated sodium channels, since the F1586C mutation in hNav1.4, which is likely involved in the high-affinity binding of local anesthetics to inactivated sodium channels (Ragsdale et al., 1996; Desaphy et al., 2012), greatly reduces sodium current inhibition by the compound, zeroing the use-dependence. Since (*R*)-31b and (*S*)-22b displayed a greater analgesic activity than sumatriptan in the hot plate test (Carocci et al., 2010), we hypothesize that the blockade of DRG sodium channels is likely involved in the mechanism of action of the bioisosteres. On an other hand, the limited analgesic potency of sumatriptan in the hot plate test may rely on its difficulty to cross the blood brain barrier, due to its negative Log *D*, and its limited activity on sodium channels. We thus propose the compounds (*S*)-22b and (*R*)-31b as potential starting compounds for the synthesis of new drugs which could be useful in the treatment of chronic pain.

## Materials and Methods

### Cell Culture and Patch-Clamp Recordings

Permanent expression of PNS hNav1.7 sodium channel subtype, skeletal muscle hNav1.4 subtype, and its F1586C mutant was obtained in human embryonic kidney (HEK293) cells as previously described (Desaphy et al., 2009, 2010).

Sodium currents (I_Na_) were recorded using the patch-clamp technique in whole-cell configuration at room temperature (21–22°C), using Axopatch 1D amplifier (Axon Instruments, Union City, CA, USA). Voltage-clamp protocols and data acquisition were performed with pCLAMP software (version 10.3, Axon Instr.) through a 12-bit A–D/D–A interface (Digidata 1440A, Axon Instr.). Patch pipettes had resistance ranging from 1 to 3 MΩ. Currents were low-pass filtered at 2 kHz (-3 dB) by the four-pole Bessel filter of the amplifier and were digitized at 10–20 kHz as previously described (Desaphy et al., 2009, 2010, 2012). In the whole-cell configuration, a 25-ms long test pulse at -30 mV was applied from the holding potential (HP) of -120 mV at a low frequency until stabilization of I_Na_ amplitude and kinetics was achieved (typically 5 min). Only those data obtained from cells exhibiting series resistance errors <5 mV were considered for analysis. Capacitance currents were partially compensated using the *ad hoc* circuit of the amplifier. Residual capacitance transients and leak currents were eliminated off-line by subtraction of the scaled passive current response recorded upon return to the HP. After reaching stable I_Na_, a maximum of two drug concentrations were tested on each cell, to minimize the possible bias due to I_Na_ run-down. Typically, a single cell experiment did not last more than 30 min, which is associated to a run-down of peak current amplitude lower than 10% (mean ± SEM, 6.2 ± 0.7%, *n* = 10). Concentration–response curves were thus drawn by combining results obtained in various cells at both stimulation frequencies and fit with Eq. 1: I_DRUG_/I_CTRL_ = 1/{1 + ([Drug]/IC_50_)^nH^}, where IC_50_ is the concentration needed to produce a 50% reduction of I_Na_ and nH is the slope factor.

To characterize the voltage dependency of steady-state channel activation, currents were evoked by 25 ms-long voltage steps ranging from -100 to +70 mV, applied in 5 mV increments every 10 s. The HP was -150 mV to be sure having all the channels available for opening. The conductance (G_Na_) values were calculated from measured peak I_Na_ currents and calculated reversal potential for sodium ions (E_Na_ = +68.4 mV) using Eq. 2: G_Na_ = I_Na_/(V-E_Na_). Resulting values for conductance were normalized to the maximal conductance and plotted as a function of voltage. The relationships were fitted using the Boltzmann Eq. 3: G_Na_/G_Na,max_ = 1/{1 + exp[(V - V_50_)/K]}, where G_Na,max_ is the maximal conductance, K is the slope factor, and V_50_ is the potential at which half of the channels are activated.

The voltage dependency of steady-state fast inactivation (channel availability) was determined using a two-pulse protocol. The HP was -160 mV; conditioning voltage steps were applied for 50 ms from -120 to -20 mV, in 10-mV increments, and followed by a test pulse at -20 mV for 20 ms. The normalized peak current amplitude measured during the test pulse was plotted as a function of the conditioning pulse voltage. The relationships were fitted using the Boltzmann Eq. 4: I_Na_/I_Na,max_ = 1/(1 + exp[(V-V_h_)/S]), where I_Na,max_ is the maximal current amplitude, S is the slope factor, and V_h_ is the potential at which half of the channels are inactivated.

### Statistical Analysis

Averaged experimental points are reported as means ± SEM from at least three patches. The fit parameters of averaged relationships are given together with the SE of the regression. Because the concentration–response curves were obtained by combining results obtained in different cells at the various concentrations, the statistical comparison of I_Na_ inhibition by the different exploratory compounds was performed at each concentrations using analysis of variance (ANOVA) followed by *ad hoc* Bonferroni’s *t*-test. On the other hand, to perform statistical analysis of (*S*)-22b effects on activation and fast inactivation, the complete voltage-dependence relationships were obtained in each cells, and the fit parameters obtained in each cells were averaged as means ± SEM from eight cells. Statistical analysis was performed using paired Student’s *t*-test. Statistical significance was defined as *p* < 0.05.

### Drugs and Solutions

The patch pipette solution contained in mM: 120 CsF, 10 CsCl, 10 NaCl, 5 EGTA, and 5 HEPES, and the pH was adjusted to 7.2 with CsOH, while the bath solution contained in mM: 150 NaCl, 4 KCl, 2 CaCl_2_, 1 MgCl_2_, 5 HEPES, and 5 glucose and pH was adjusted to 7.4 with NaOH. Sumatriptan and its analogs were synthesized in our laboratories as previously described in details (Carocci et al., 2010). Sumatriptan was synthesized as succinate salt, while (*R*)-31b, 20b and (*S*)-22b as hydrochloride salts. Mexiletine was purchased from Sigma–Aldrich (Milan, Italy). All the compounds were solubilized directly in bath solution to obtain the desired final concentrations. To test drugs, the patched cells were first exposed to a continuous stream of control bath solution and later exposed to a continuous stream of drug-supplemented bath solution, flowing out from a plastic capillary.

## Results

**FIGURE 2:**
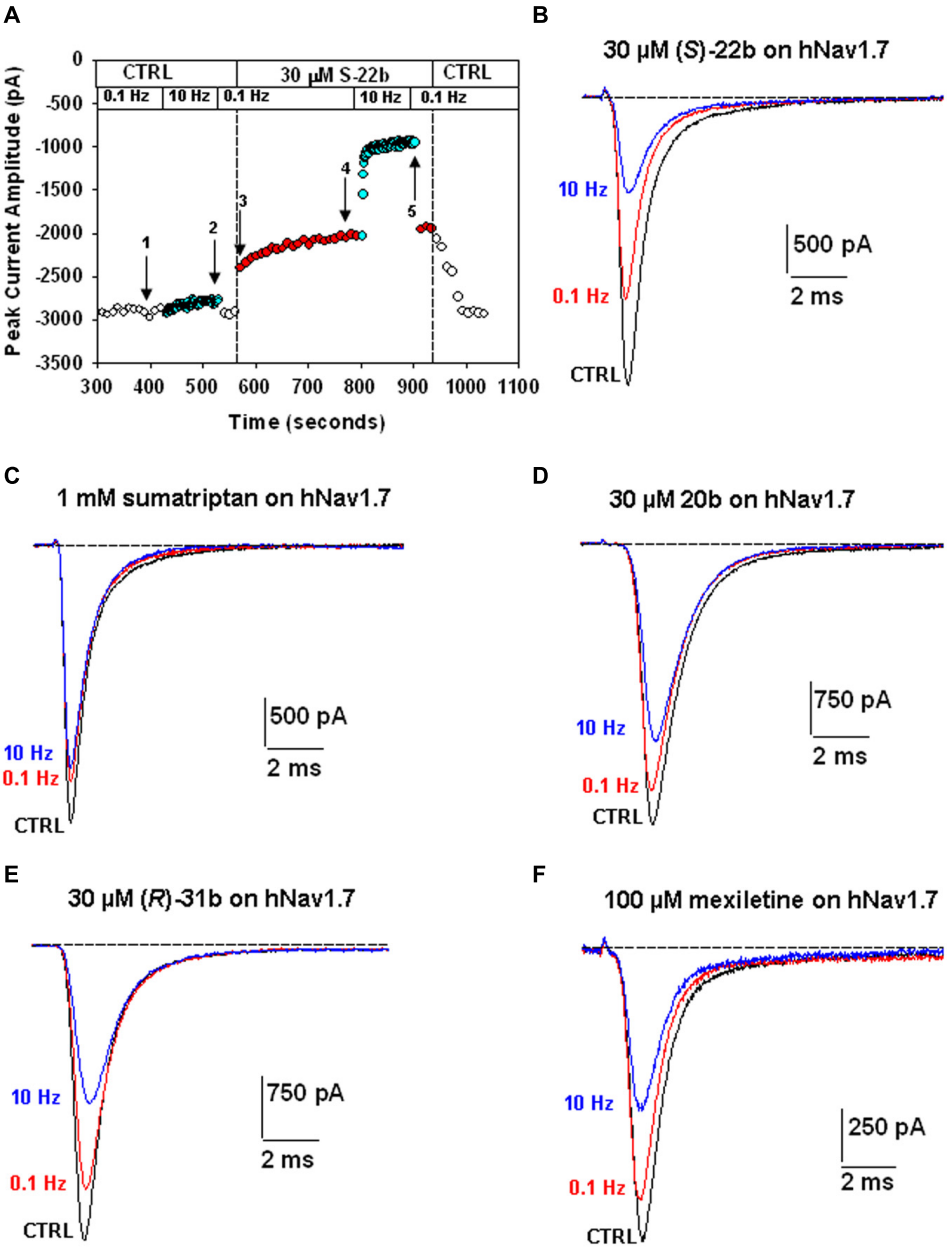
**Effects of exploratory compounds on hNav1.7 channels at 0.1 Hz and 10 Hz, to determine tonic and phasic block, respectively. (A)** Time course of peak sodium current amplitude in a representative HEK293 cell permanently transfected with hNav1.7, before and during application of 30 μM (*S*)-22b, and after drug washout. The membrane was held at –120 mV and depolarized to –30 mV for 25 ms at 0.1 or 10 Hz stimulation frequency. The numbered arrows correspond to effects described in the text. **(B)** Typical sodium current traces recorded in the same cell as in **(A)**, which were obtained from the average of three records obtained at steady state in control at 0.1 Hz (corresponding to arrow 1 in **A**), and in presence of 30 μM (*S*)-22b at 0.1 Hz (arrow 4 in **A**) and 10 Hz (arrow 5 in **A**). **(C–F)** Representative sodium current traces recorded as in **(A,B)**, after application of 1 mM sumatriptan, 30 μM of 20b and (*R*)-31b compounds, and 100 μM mexiletine.

**FIGURE 3:**
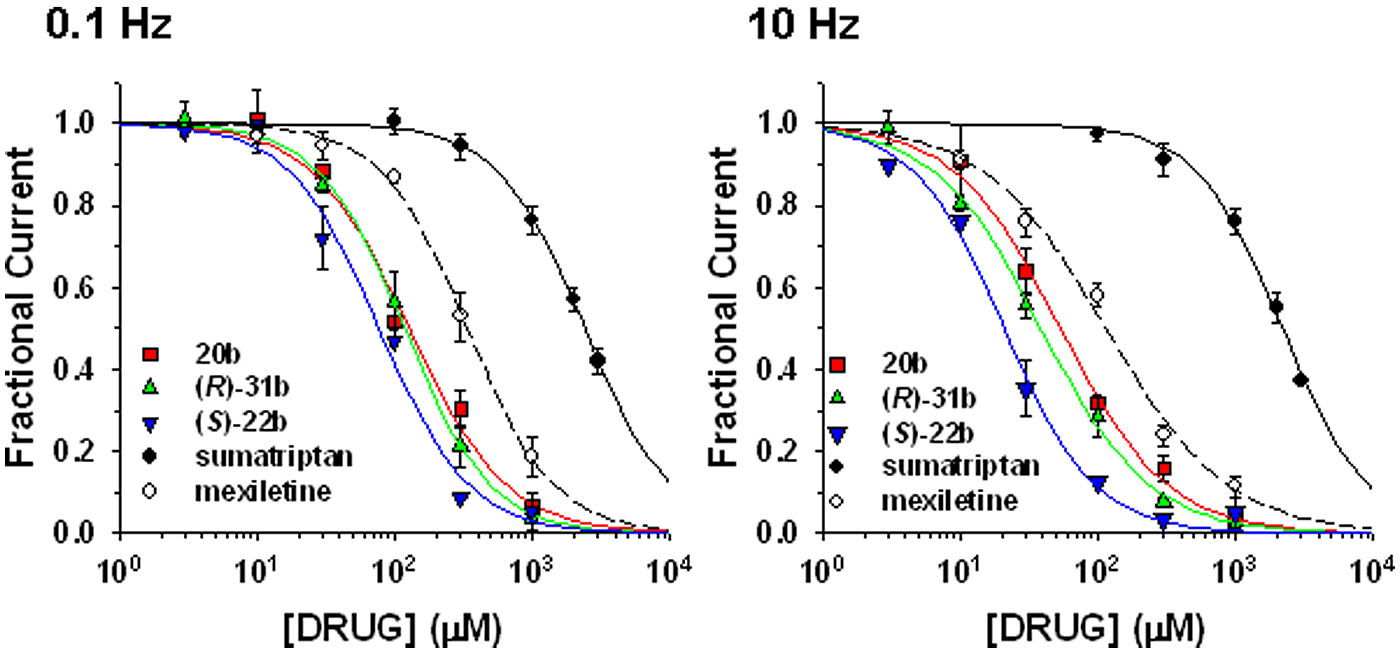
**Concentration–response relationships for mexiletine, sumatriptan, and its three analogs on hNav1.7 channels**. The curves were constructed at 0.1 and 10 Hz frequency stimulations from peak amplitude values of sodium currents recorded as described in **Figure 2**, and fitted with Eq. 1 given in the “Materials and Methods” section. Each data point is the mean ± SEM of at least three cells. The fit parameters together with the SE of the regression are given in **Table 1**. Statistical analysis is reported in **Table 2**.

Sumatriptan and its analogs, as well as mexiletine, were tested on hNav1.7 channels permanently expressed in HEK293 cell line. Sodium currents were recorded using the patch-clamp technique in whole-cell configuration, before and after the application of drugs. The currents were elicited by depolarizing the cell membrane from the HP of -120 to -30 mV at two stimulation frequencies, 0.1 Hz and 10 Hz, to determine both tonic and phasic blocks. **Figure 2A** illustrates the time course of Nav1.7 current amplitude in a representative cell during application of 30 μM (*S*)-22b. Before drug application, peak current amplitude was stable at 0.1 Hz frequency (open circles, first arrow in **Figure 2A**). Increasing the stimulation frequency to 10 Hz slightly reduced current amplitude by less than 10% (cyan circles, second arrow in **Figure 2A**; mean ± SEM, 9.8 ± 0.8%, *n* = 56 cells). Application of the drug reduced the peak current amplitude by 17.9% (17.6 ± 3.9%, *n* = 3) on first voltage step with respect to control (third arrow in **Figure 2A**) and further gradually reduced sodium current at 0.1 Hz (red circles). At the steady-state reached after about 3 min, the peak current amplitude was 69.6% of control (fourth arrow in **Figure 2A**; 71.9 ± 4.8%, *n* = 3). Applying 10 Hz stimulation, the drug induced a huge and rapid use-dependent inhibition of sodium currents by 53% (59.3 ± 6.7%, *n* = 3). At steady state, the peak current amplitude in presence of the drug was 34.2% of control current measured at 10 Hz (fifth arrow in **Figure 2A**; 35.1 ± 6.6%, *n* = 3). The effects of 10 Hz stimulation frequency and drug were fully reversible, since the current amplitude after washout was 93.3% of control current recorded before drug application (90.9 ± 1.9%, *n* = 3). **Figure 2B** shows representative sodium current traces recorded in the same cell as in **Figure 2A** at steady-states before and after the application of 30 μM (*S*)-22b at both stimulation frequencies. Representative current traces obtained using the same protocols are shown for mexiletine, sumatriptan, and its derivatives. Sumatriptan showed a lower affinity to the channel compared to all other compounds: 1 mM sumatriptan exerted 23.6 ± 3.2% (*n* = 3) inhibition at 0.1 Hz and 23.9 ± 3.1% (*n* = 3) inhibition at 10 Hz. A 30 μM concentration of 20b and (*R*)-31b produced a similar tonic block (11.6 ± 0.8% (*n* = 4) and 14.9 ± 2.4% (*n* = 3), respectively), while use-dependence was accentuated with 36.0 ± 3.1% (*n* = 4) and 44.4 ± 3.2% (*n* = 3) inhibition at 10 Hz, respectively. Mexiletine at the concentration of 100 μM produced an effect similar to 30 μM (*R*)-31b, with 13.2 ± 1.5% and 42.0 ± 2.8% (*n* = 4) inhibition at 0.1 and 10 Hz, respectively. As for (*S*)-22b, the effects of all drugs were quite reversible (not shown). The concentration–response curves (**Figure 3**) were constructed from the effects measured in different cells at the various concentrations using similar protocols and were fitted with Eq. 1 (*see* Materials and Methods) to determine the IC_50_ values (**Table 1**). Statistical analysis was performed using ANOVA followed by *ad hoc* Bonferroni’s *t*-test to compare the percentage of inhibition of sodium currents exerted by the various drugs at 30, 100, 300, and 1000 μM (**Table 2**). Sumatriptan appeared as a very weak blocker of hNav1.7 sodium channels and did not display any use-dependence. In contrast, the three analogs blocked hNav1.7 currents at concentrations more similar to those of the well-known sodium channel blocker, mexiletine. Indeed, 20b, (*R*)-31b, and (*S*)-22b were 2.6, 2.9, and 4.4 fold more potent at 0.1 Hz than mexiletine. The compound (*S*)-22b was significantly more potent than the others compounds at both 0.1 and 10 Hz. The use-dependent behavior of drugs, expressed as the ratio of IC_50_ values calculated at 0.1 and 10 Hz, were very similar for mexiletine, (*S*)-22b, and (*R*)-31b.

**Table 1:** Comparison between half-maximum inhibitory concentrations of sumatriptan and its analogs on hNav1.7 sodium channels and of (*S*)-22b on wild-type and mutated hNav1.4.

Drug	0.1 Hz		10 Hz		0.1 Hz/10 Hz IC_50_ ratio
	
	IC_50_ (μM)	nH	IC_50_ (μM)	nH	
***hNav1.7***
Sumatriptan	2399 ± 56	1.4 ± 0.1	2187 ± 106	1.4 ± 0.1	1,1
20b	127 ± 16	1.2 ± 0.2	54 ± 4	1.1 ± 0.1	2,4
(*R*)-31b	118 ± 5	1.4 ± 0.1	39 ± 3	1.1 ± 0.1	3,0
(*S*)-22b	77 ± 10	1.3 ± 0.2	21 ± 2	1.3 ± 0.1	3,7
Mexiletine	338 ± 18	1.4 ± 0.1	114 ± 12	1.0 ± 0.1	3,0
***hNav1.4***
(*S*)-22b	51 ± 2	1.4 ± 0.1	12 ± 1	1.3 ± 0.1	4,3
***hNav1.4 – F1586C***
(*S*)-22b	215 ± 5	1.4 ± 0.1	175 ± 9	1.4 ± 0.1	1,2

*The values were obtained from the fit of the concentration–response curves illustrated in **Figures 2** and **5**. The half-maximum inhibitory concentration (IC_*50*_) and the slope factor (nH) are given as the fit value ± SE of the fit*.

**Table 2:** Statistical analysis (ANOVA) of hNav1.7 sodium channel inhibition by exploratory compounds at the holding potential (HP) of –120 mV, at 0.1 Hz **(A)** and 10 Hz **(B)** stimulation frequencies.

	Sumatriptan		Mexiletine		20b		(*R*)-31b	
**A: 0.1 Hz**								
Mexiletine		yes			
	yes				
20b		yes		yes		
	yes		yes			
(*R*)-31b		yes		yes		ns	
	yes		yes		ns		
(*S*)-22b		yes	yes	yes	yes	ns	yes	ns
	yes		yes		yes		yes	
**B: 10 Hz**								
Mexiletine		yes			
	yes				
20b		yes	yes	yes		
	yes		yes			
(*R*)-31b		yes	yes	yes		ns	
	yes		yes		yes		
(*S*)-22b		yes	yes	yes	yes	yes	yes	yes
	yes		yes		yes		ns	

*The percentages of inhibition of sodium channels by the various drugs at each concentration, as reported in concentration–response relationships (**Figure 3**), were compared using ANOVA followed by ad hoc Bonferroni’s t-test. The significance is reported as “yes” (at least p < 0.05) or “ns” (not significant) in cyan cells (for comparison at 30 μM), yellow cells (100 μM), orange cells (300 μM), and pink cells (1000 μM). Note that sumatriptan was not tested at 30 μM (gray cells)*.

**FIGURE 4:**
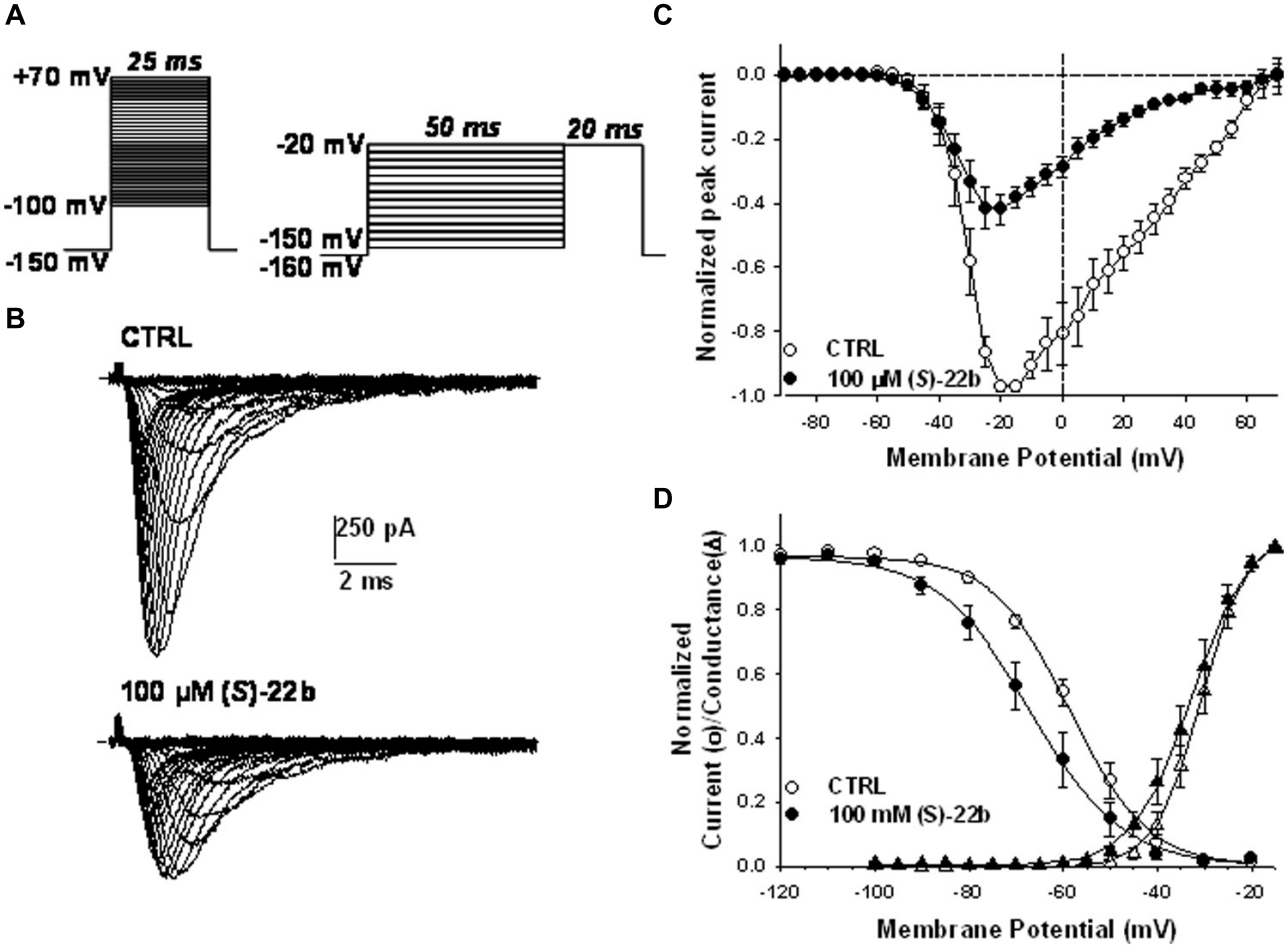
**Effects of 100 μM (*S*)-22b on the voltage dependence of hNav1.7 channels. (A)** Left protocol, to obtain the current–voltage (I–V) and activation relationships, the membrane was held at –150 mV and depolarized by successive 25 ms-long voltage steps ranging from –100 to +70 mV applied in 5-mV increments every 10 s. Right protocol, to obtain the voltage dependence of steady state availability (fast inactivation), the membrane was held at –160 mV, and conditioning 50 ms-long voltage steps were applied from –120 to –20 mV in 10-mV increments, before to apply a 20 ms-long test pulse at –20 mV. **(B)** Families of sodium currents recorded in a representative cell using the left protocol shown in **(A)**, in control conditions (CTRL) and at steady state during the application of 100 μM (*S*)-22b. **(C)** Averaged I–V relationships obtained using the left protocol shown in **(A)**. In each tested cell, the peak current amplitude measured in CTRL and in presence of 100 μM (*S*)-22b was normalized with respect to maximal peak current amplitude recorded in CTRL. Each point value is the mean ± SEM calculated from eight cells. **(D)** The voltage dependence of activation (triangles) was drawn by reporting the normalized conductance, calculated from the I–V curves with Eq. 2, as a function of test pulse voltage and fitted with Eq. 3. The voltage dependence of channel availability (circles) was drawn by reporting the normalized peak sodium current amplitude measured during the test pulse as a function of conditioning pulse voltage (right protocol in **A**), and fitted with Eq. 4. Each point value is the mean ± SEM calculated from eight cells. The averaged fit parameters, calculated from the relationships obtained in each cells, are reported in the text.

Effects of the more potent (*S*)-22b were examined on the voltage dependence of Nav1.7 activation and fast inactivation. The current–voltage relationship was determined in eight cells expressing hNav1.7 channels, before and after application of 100 μM (*S*)-22b, by depolarizing the membrane from –100 to +70 mV for 25 ms, in 5 mV increments, every 10 s (**Figure 4A**, left protocol). The HP was –150 mV. A representative example of current traces recorded in the absence and presence of (*S*)-22b is shown in **Figure 4B**. The averaged I–V relationships (*n* = 8) are shown in **Figure 4C**. The drug did not change the voltage to elicit maximal current amplitude (between –20 and –15 mV), but drastically reduced current amplitude at voltage superior to –30 mV. The voltage dependence of steady-state channel activation was obtained from the I–V curves and fit with Eq. 3 (**Figure 4D**). The half-maximum activation potential (V_50_) and the slope factor (K) were not significantly modified by the drug (V_50_ = –30.7 ± 1.5 mV and K = –4.2 ± 0.3 mV in CTRL; V_50_ = –33.5 ± 1.8 mV and K = –4.8 ± 0.3 mV in presence of drug; mean ± SEM, *n* = 8; not significant with paired Student’s *t*-test). The voltage dependence of steady-state fast inactivation was determined using the two-pulse protocol shown in **Figure 4A** (right). The HP was –160 mV; cell membrane was depolarized for 50 ms from -120 to -20 mV, in 10-mV increments; a test pulse was applied at -20 mV for 20 ms. The relationships were constructed by reporting the normalized peak current amplitude measured during the test pulse as a function of the prepulse test voltage. The averaged relationships (*n* = 8) and Boltzmann fits with Eq. 4 are shown in **Figure 4D**. The drug induced a significant 8.8 mV negative shift of the half-maximum inactivation potential (V_h_) from -57.5 ± 1.8 to -66.3 ± 3.3 mV (mean ± SEM, *n* = 8; *P* < 0.01 with paired Student’s *t*-test). The slope factor (S) was not significantly altered by the drug (S = 8.1 ± 0.5 mV in CTRL and 8.3 ± 0.3 mV in presence of drug, *n* = 8).

**FIGURE 5:**
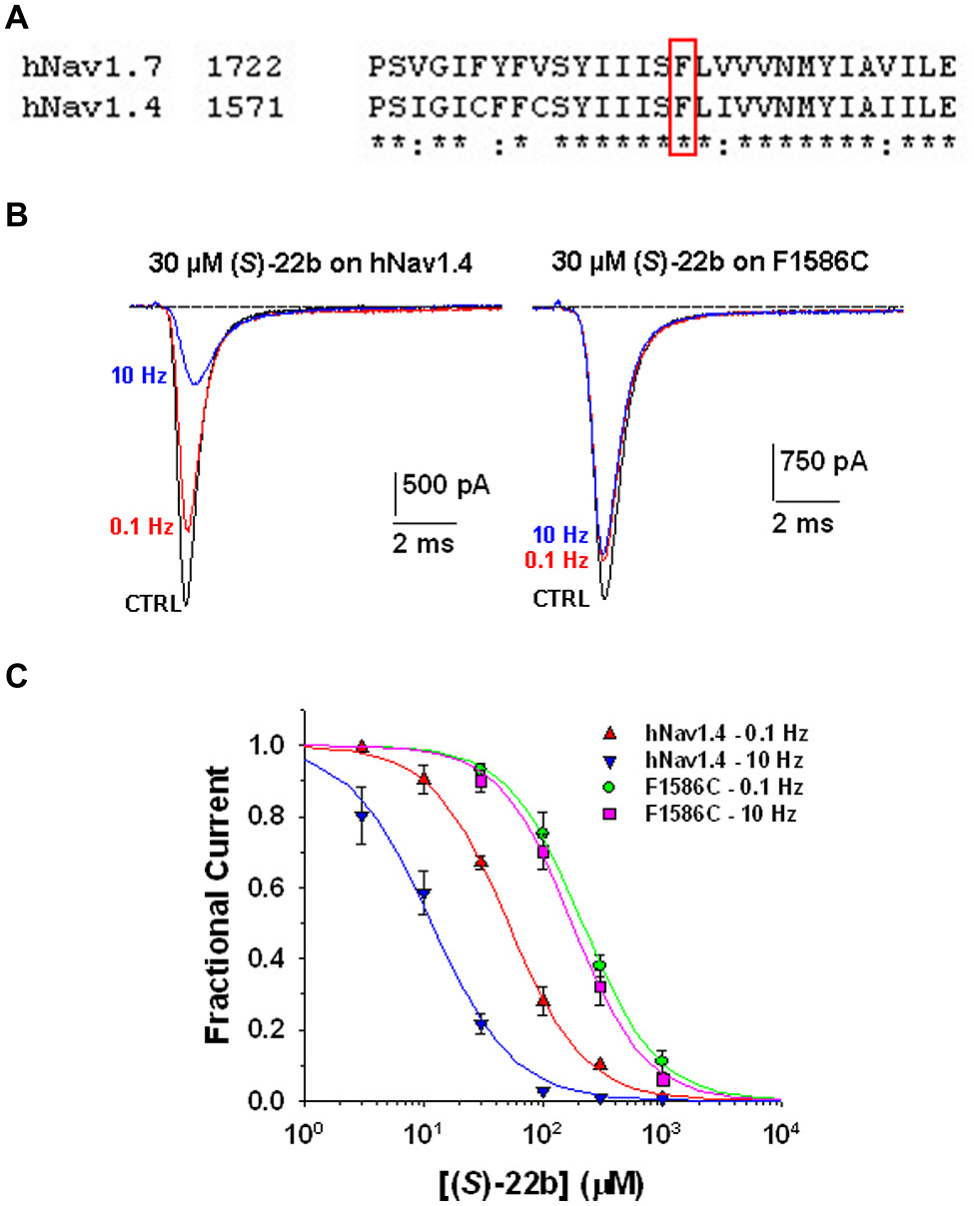
**Effects of (*S*)-22b on wild-type hNav1.4 channels and F1586C mutant. (A)** Amino acid alignment of hNav1.7 and hNav1.4 using Clustal W 2.0 shows high conservation of the putative local anesthetic receptor located within the sixth segment of domain IV. The phenylalanine residue in red box (F1586 in hNav1.4 and F1737 in hnav1.7) is thought to be critical for the binding of local anesthetics to inactivated sodium channels. **(B)** Representative traces of sodium currents recorded as in **Figure 2** in HEK29 cells permanently transfected with wild-type hNav1.4 or F1586C hNav1.4 mutant. The inhibitory effects of 30 μM (*S*)-22b were greatly reduced in the F1586C mutant. **(C)** Concentration–response relationships for (*S*)-22b at 0.1 and 10 Hz stimulation frequencies were obtained as in **Figure 3**, and fitted with Eq. 1. Each data point is the mean ± SEM of at least three cells. At both stimulation frequencies, the inhibition of F1586C channels by (*S*)-22b was significantly minor with respect to that of WT channels at 30, 100, 300, and 1000 μM (at least *P* < 0.05 with unpaired Student’s *t*-test). The fit parameters together with the SE of the regression are given in **Table 1**.

Use-dependent block and shift of fast inactivation voltage dependence are characteristics of many clinically used sodium channel blockers, including the antiarrhythmic mexiletine. These drugs are thought to bind the local anesthetic molecular receptor located within the ion-conducting pore of sodium channels, involving amino acid residues of the transmembrane S6 segment of domain IV. The putative local anesthetic receptor is very well conserved among sodium channel subtypes, including hNav1.7 and the skeletal muscle hNav1.4 subtype (**Figure 5A**). A conserved phenylalanine residue (Phe1586 in hNav1.4) is especially critical for high-affinity binding to inactivated channels and use-dependent block (Ragsdale et al., 1996; Desaphy et al., 2009, 2012). To verify whether (*S*)-22b binds to the same site as local anesthetics, we thus tested the drug on wild-type and mutated (F1586C) hNav1.4, permanently transfected in HEK293 cells (Desaphy et al., 2010, 2012). Representative hNav1.4 and F1586C sodium current traces recorded before and after application of 30 μM (*S*)-22b are shown in **Figure 5B**. The (*S*)-22b compound resulted less potent on F1586C mutant, as well as less use-dependent, with respect to wild-type channels. At 30 μM concentration, (*S*)-22b exerted a 25% blocking activity at 0.1 Hz and a 74% blocking activity at 10 Hz on WT hNav1.4, compared to 13% at 0.1 Hz and 16% at 10 Hz on F1586C. The dose–response curves (**Figure 5C**) show the greatly reduced potency of (*S*)-32b and the total lack of use-dependence on F1586C at 0.1 Hz. Fit parameters are given in **Table 1**. This result indicate that Phe1586 is critically involved in the high-affinity binding of (*S*)-22b to inactivated sodium channels.

## Discussion

Using the patch-clamp technique to evaluate the effects of sumatriptan and its analogs on peripheral neuronal sodium channels, we observed that sumatriptan is a weak sodium channel blocker, since the concentration necessary to produce a significant sodium current reduction is greater than 100 μM, whereas the therapeutic blood concentration range is 61–203 nM (Schulz and Schmoldt, 2003). In addition, sumatriptan did not display any use-dependent behavior. In contrast, we observed that three recently reported analogs of sumatriptan exert a substantial block of hNav1.7 sodium currents, greater than the one exerted by the well-known sodium channel blocker mexiletine. The compounds were 2.5-to-4 fold more potent than mexiletine in blocking sodium currents at 0.1 and 10 Hz stimulation, displaying a similar use-dependent behavior. The most potent (*S*)-22b compound induced a significant negative shift of voltage dependence of steady-state inactivation, as many local anesthetic-like drugs. Accordingly, we demonstrated that (*S*)-22b binds sodium channels at the conserved local anesthetics receptor site, since it shows a reduced potency and a total absence of use-dependence when applied to the hNav1.4 F1586C mutant compared to wild-type hNav1.4 channel. Thus, (*S*)-22b could be considered as a true local anesthetic-like drug.

Probably, the chemical structure of sumatriptan – containing a sulfonamide group, an aromatic ring consisting in an indolic ring, and an ethylic chain – impedes high affinity binding to sodium channels. It should be noted that sumatriptan is little lipophilic, displaying a negative Log *D*-value at pH 7.4 (Rance et al., 1997), which likely limits its inhibitory activity on sodium channels (Desaphy et al., 2012). In contrast, the sumatriptan analogs include a naphthyloxy group linked to a protonable amine through an alkyl chain, which are pharmacophoric elements commonly found in other known sodium channel blockers, such as propranolol (Desaphy et al., 2003; Wang et al., 2010a). The three compounds show a Log *D*-value greater than unity at pH 7.4 (Carocci et al., 2010). Like propranolol, the sumatriptan analogs are more potent than mexiletine, most probably because the naphthyl moiety increases steric hindrance and lipophilia with respect to the xylyl one in mexiletine (Desaphy et al., 2012). Compared to 20b, the constraint of the nitrogen atom into a pyrrolidine ring as in (*R*)-31b slightly improves use-dependence, in agreement with previous studies, in which we demonstrated that the constriction of the N atom of tocainide in a pyrrolidine enhances binding affinity to sodium channels in the inactivated state (De Luca et al., 2003). Interestingly, the introduction of a methyl in C2 close to the oxygen, as in (*S*)-22b, allows a greater gain of blocking activity at both stimulation frequencies.

Using the hot plate test on mice, we have previously demonstrated that sumatriptan and its analogs induce analgesia *in vivo* (Carocci et al., 2010). At the dose of 30 mg/kg, (*R*)-31b showed the greatest analgesic efficacy lasting for 45 min, but (*S*)-22b displayed significant analgesia lasting at least for 75 min. At the same concentration, the analgesic efficacy of sumatriptan and 20b were more modest. At 10 mg/kg, only (*S*)-22b exerted significant analgesia, appearing as the most potent compound. It should be noted that the doses tested in this previous study are quite high (Carocci et al., 2010). While sumatriptan was shown to exert antinociceptive effects in a rat model of trigeminal neuropathic pain at the clinically relevant dose of 0.1 mg/kg s.c. (Kayser et al., 2002), it remains to be verified whether (*S*)-22b may produce analgesia at lower doses.

In the same study, we also demonstrated that the chemical maneuvers on sumatriptan analogs resulted in significant alterations of their serotonergic profile: Compared to sumatriptan, 20b displayed a 2.5- and 3.6-fold reduced affinity toward 5HT_1B_ and 5HT_1D_ receptors, respectively; (*R*)-31b conserved agonism only toward 5HT_1D_ receptor (twofold reduction compared to sumatriptan) but showed a 13-fold reduced affinity toward 5HT_1B_; in contrast, (*S*)-22b was associated with a drastically reduced affinity to both serotonergic receptors (eightfold reduction toward 5HT_1B_ and 360-fold reduction toward 5HT_1D_). Thus the analgesic activity measured in the mouse was not correlated to binding affinities to serotonin receptors, suggesting that at least part of the analgesic effect of the new compounds is not mediated by serotonergic agonism.

**FIGURE 6:**
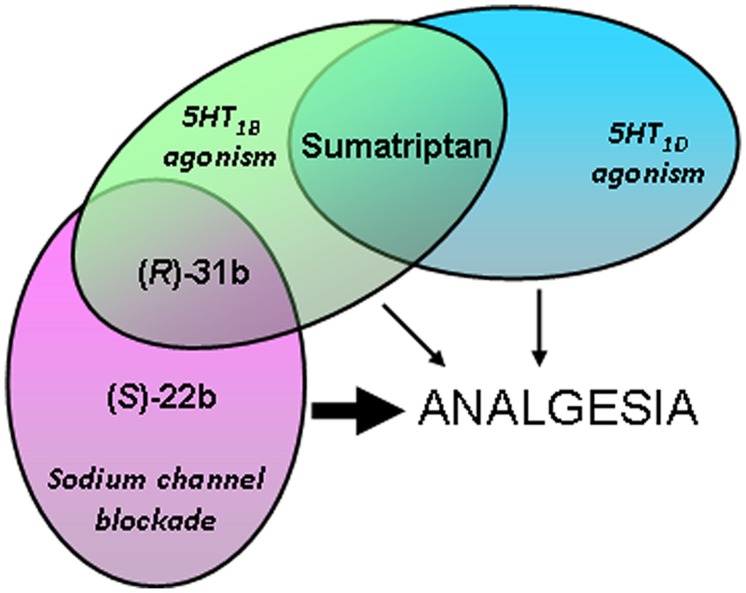
**Cartoon depicting hypothesis regarding pharmacological actions of exploratory compounds displaying analgesic activity *in vivo*, based on the results obtained in the present and previous studies (Carocci et al., 2010).** The thickness of the arrows indicates the intensity of the analgesic activity exerted by the compounds.

Combining the pharmacological results obtained in the present study with those obtained *in vivo* and in binding affinity studies (**Figure 6**), we hypothesize that the analgesic activities of (*S*)-22b and (*R*)-31b result, at least in part, from their ability to block sodium channels at the higher frequency. In addition, 5HT_1D_ serotonergic agonism may also contribute to (*R*)-31b analgesic activity. On the other hand, the weak *in vivo* analgesic profile showed by sumatriptan may rely in part on its weak activity on sodium channels. Another likely important limiting factor for sumatriptan analgesic effects is its low lipophilia, which may reduce its permeability across the blood brain barrier. Indeed, intrathecal administration of sumatriptan low doses was shown to reduce persistent inflammatory pain in mice (Nikai et al., 2008). Thus the increase lipophilia of (*S*)-22b and (*R*)-31b might be advantageous for reaching significant analgesia *in vivo*. Finally, we suggest that 20b displays an analgesic activity comparable to sumatriptan, because it may not gain enough use-dependent sodium channel blocking activity. It is worth to note that the apparent affinity for sodium channel blockade may appear lower than the binding affinity to serotonergic receptors, but the patch-clamp experiments likely underestimate the actual affinity for use-dependent I_Na_ inhibition in a physiopathological condition, which is characterized by less negative resting membrane voltage and high-frequency discharges of action potentials.

## Conclusion

Our results suggest that (*S*)-22b and (*R*)-31b could be considered as efficient sodium channel blockers associated with a significant antihyperalgesic profile. It should be kept in mind that, as most sodium channel blockers, these compounds may act on various sodium channel isoforms (at least hNav1.4 in this study) and that use-dependence is a critical issue for safety. It should be mentioned that targeting either specific isoforms or activities of sodium channels (or both), all may be valid strategies to develop treatment of pain syndromes (Theile and Cummins, 2011). Although maintaining a residual 5HT_1D_ activity, (*R*)-31b probably lacks of clinically relevant activity on 5HT_1B_ receptors. It is noteworthy that 5HT_1B_ agonism may be associated with the cardiovascular side effects usually observed with triptans, which limits their clinical use in patients who suffer from cardiac diseases. Thus, (*S*)-22b and (*R*)-31b could represent interesting lead compounds for the synthesis of new analgesic drugs the mechanism of action of which can involve potent and use-dependent blockade of sodium channels or a dual action on sodium channels and 5HT_1D_ receptors.

## Conflict of Interest Statement

The authors declare that the research was conducted in the absence of any commercial or financial relationships that could be construed as a potential conflict of interest.
